# COVID-19 fear and its associated correlates among type-2 diabetes patients in Bangladesh: A hospital-based study – CORRIGENDUM

**DOI:** 10.1017/gmh.2023.60

**Published:** 2023-10-16

**Authors:** Suvasish Das Shuvo, Md. Toufik Hossen, Md. Sakhawot Hossain, Asma Khatun, Sanaullah Mazumdar, Md. Riazuddin, Deepa Roy

The authors apologise that a number of numerical values within *The section Factors associated with the COVID-19 fear* and in table 2 were incorrect.

The correct section and tables are below:

Table 2 presents the adjusted association between COVID-19 fear with demographic and healthcare characteristics. In the adjusted regression model, gender, age, occupation, residence, physical activity, smoking, DDS score, limited self-care practice, unaffordable medicine, medicine shortage, a close friend or family member diagnosed with COVID-19, and financial problem during COVID-19 were significantly associated with fear (FCV-19S). Females had approximately 4 times more fear of COVID-19 compared to males (OR = 3.83, 95% CI: 1.83–6.38), whereas ages between 50 and 64 years and above 65 years also showed 1.28 times and 1.51 times more fear than their counterparts (OR = 1.28, 95% CI: 1.12–2.46; OR: 1.51, 95% CI: 1.09–3.16). Regarding occupation, unemployed patients, and nonmanual workers were 2.47 times and 2.31 times (OR: 2.47, 95% CI: 1.76–4.17); OR: 2.31, 95% CI: 1.82–4.54) more fearful compared to the manual worker. It is also observed that patients residing in urban areas were 2.51 times (OR: 2.51, 95% CI: 1.24–4.16) more likely to fear compared with peers residing in rural areas. Moreover, type-2 diabetes patients undertaking a recommended level of MVPA (more than thrice to every day) had 0.66 times (OR: 0.66, 95% CI: 0.34–0.82) lower chances of being fear compared with peers performing less than the recommended level of physical activity. Additionally, the odds of being fear was 3.34 times (OR: 3.34, 95% CI: 1.42–5.32) and 1.21 times (OR: 1.21, 95% CI: 1.13–1.95) higher among current smoker and had low DDS, respectively, compared with their nonsmoker and high DDS counterparts. Again, those who had anxiety and comorbidity were 1.66 times and 1.43 times more likely to fear as compared to their counterparts (OR 1.66, CI: 1.27–3.53; OR 1.43, CI: 1.19–2.24). On the other hand, those who had limited self-care practice and unaffordable medicine were 3.49 times and 1.13 times higher odds (OR 3.49, CI: 1.27–5.76; OR 1.13, CI: 1.03–1.92) of being fear as compared to those peers. Lastly, we found type-2 diabetes patients who had medicine shortages, a close friend or family member diagnosed with COVID-19, and financial problems during COVID-19 were almost 2.27 times, 3.83 times, and 2.92 times higher risk of being fear as compared to their peers (OR: 2.27, CI: 1.24–4.16; OR: 3.83, CI: 1.42–6.35; OR: 2.92, CI: 1.54–4.58), respectively.

**Table 2.** Association between COVID-19 fear with demographic and healthcare characteristics

Variables	Category	OR (95% CI)	*p*-value
Gender	Male	1	
	Female	3.83 (1.83–6.38)	0.004
Age	Below 35 years	1	
	35–49 years	0.77 (0.32–1.89)	0.576
	50–64 years	1.28 (1.12–2.46)	0.005
	Above 65 years	1.51 (1.09–3.16)	0.041
Education	Graduates	1	
	HSC	0.76 (0.25–2.27)	0.559
	Secondary	0.43 (0.17–1.04)	0.056
	Primary	1.50 (1.18–2.30)	0.013
	Illiterate	1.78 (1.28–2.79)	0.021
Occupation	Manual worker	1	
	Nonmanual worker	2.31 (1.82–4.54)	0.005
	Unemployment or retired	2.47 (1.76–4.17)	0.001
Family monthly income	>20,000 BDT	1	
	15,000–20,000 BDT	1.39 (1.26–2.51)	0.029
	10,001–15,000 BDT	0.85 (0.46–1.53)	0.552
	<10,000 BDT	0.95 (0.45–2.01)	0.884
	Depend on other	0.26 (0.09–0.75)	0.013
Residence	Rural	1	
	Urban	2.51 (1.24–4.16)	0.021
Family history of diabetes	No	1	
	Yes	1.37 (0.85–2.13)	0.22
Physical exercise	No	1	
	Yes	0.37 (0.17–0.76)	0.039
MVPA	Less than the recommended level	1	
	Recommended level	0.66 (0.34–0.82)	0.019
Smoking habit	Nonsmoker	1	
	Ex-smoker	0.33 (0.15–0.70)	0.005
	Current smoker	3.34 (1.42–5.32)	0.030
BMI	Healthy weight	1	
	Underweight	0.39 (0.13–1.10)	0.079
	Overweight	0.70 (0.40–1.21)	0.219
	Obese	0.61 (0.33–1.12)	0.112
DDS	High	1	
	Moderate	0.72 (0.43–1.19)	0.208
	Low	1.21 (1.13–1.95)	0.001
Anxiety	No	1	
	Yes	1.66 (1.27–3.53)	0.015
Comorbidity	No	1	
	Yes	1.43 (1.19–2.24)	0.025
Read or listened to news about the COVID-19	No	1	
	Sometimes	3.15 (1.53–5.78)	0.002
	Always	2.07 (1.13–3.06)	0.024
Transport difficulties	No	1	
	Yes	1.52 (0.27–2.98)	0.244
Limited self-care practice	No	1	
	Yes	3.49 (1.27–5.76)	0.015
Delayed care seeking	No	1	
	Yes	0.93 (0.51–1.77)	0.991
Unaffordable medicine	No	1	
	Yes	1.13 (1.03–1.92)	0.038
Medicine shortage	No	1	
	Yes	2.27 (1.24–4.16)	0.008
Staff shortage	No	1	
	Yes	1.31 (0.51–3.39)	0.625
Decreased inpatient capacity	No	1	
	Yes	0.98 (0.46–2.11)	0.968
A close friend or family member diagnosed with COVID-19	No	1	
	Yes	3.83 (1.42–6.35)	0.008
Financial problems during COVID-19	No	1	
	Yes	2.92 (1.54–4.58)	0.007

BDT, Bangladeshi taka; BMI, body mass index; DDS, dietary diversity score; MVPA, moderate to vigorous physical activity; OR, odds ratios

The article has been updated.
